# Community-Based Lung Cancer Screening Program Structure, Quality, and Barriers: The Struggle for Implementation

**DOI:** 10.1155/carj/9683951

**Published:** 2025-03-21

**Authors:** Candice L. Wilshire, Kerrie E. Buehler, Claire A. Henson, Christopher R. Gilbert, Jed A. Gorden

**Affiliations:** ^1^Department of Thoracic Surgery and Interventional Pulmonology, Swedish Medical Center, Seattle, Washington, USA; ^2^Department of Medicine, Division of Pulmonary, Critical Care, Allergy, and Sleep Medicine, Medical University of South Carolina, Charleston, South Carolina, USA

## Abstract

**Objectives:** Recommendations for programmatic components for lung cancer screening programs (LCSPs) have been published; however, adoption within LCSPs has not been mandated and implementation requires resources. We aimed to determine the presence of recommended structural and quality elements within LCSPs and determine barriers to performing LCS within a community-based, multistate healthcare network.

**Methods:** We conducted a cross-sectional study using two structured interviews within a community-based healthcare network between 1 June 2018 and 31 July 2020. Two separate interviews were created, one delivered to LCSP navigators to determine the presence of recommended structural and quality elements within LCSPs and one delivered to imaging center administrators to determine barriers to LCS implementation.

**Results:** Of the 22 LCSPs, 20 (91%) were decentralized and 2 (9%) centralized. Three (14%) utilized standardized shared decision-making tools and 13 (59%) a multidisciplinary nodule review. Of the 21 (95%) LCSPs who collected information for external purposes, 9 (43%) collected it manually. Ten (45%) utilized a standard procedure for smoking cessation, and 5 (23%) had Certified Tobacco Treatment Specialists. Of the 31 affiliated imaging sites not associated with a LCSP, 8 (26%) were performing LCS. While 19 (61%) sites had the resources to fulfill or maintain an increase in LCS orders, lack of resources was the predominant (11, 35%) barrier to implementing a LCSP.

**Conclusions:** A wide variation in the structure, quality, and resource allocation was identified within the network of LCSPs. Further research identifying the implications this variation has on outcomes, operational cost, and experience may shed light on whether stringent program quality control is needed.

## 1. Introduction

Globally, lung cancer is the leading cause of cancer-related deaths, accounting for approximately 18% of all cancer deaths in 2020, with an estimated 1.8 million fatalities annually [1]. In the United States, lung cancer remains the second most commonly diagnosed cancer but is the leading cause of cancer mortality, responsible for over 127,000 deaths in 2023 alone [2]. Lung cancer's high mortality rate is primarily attributed to late-stage diagnoses, as over 74% of the cases in the United States are diagnosed at a late stage where regional and distant metastases have occurred and curative treatment options are limited and less effective [3]. This underscores the critical role of early detection through lung cancer screening.

Lung cancer screening programs (LCSPs) have proliferated across the country since publication of the National Lung Screening Trial (NLST) in 2011, the initial United States Preventive Services Task Force (USPSTF) recommendation statement in 2014, and the Center for Medicare & Medicaid Services (CMS) coverage decision in 2015 [4–7]. More recently, the 10-year Multicentric Italian Lung Detection (MILD) trial and the Nederlands-Leuvens Longkanker Screenings Onderzoek (NELSON) trial have confirmed the reduction in lung cancer-specific mortality with low-dose computed tomography (LDCT), and the USPSTF have expanded their recommended LCS criteria [8–11]. As of January 2025, there were 1367 centers across the United States that were accredited with the American College of Radiology (ACR) as a designated LCS center and had at least one accredited CT unit that met the minimum requirements for acceptable LCS performance [12].

In 2015, the American Thoracic Society (ATS) and the American College of Chest Physicians (ACCP) published their recommendations for components necessary for a high quality LCSP to standardize programs nationally [13, 14]. Nine essential elements were identified, and twenty-one policy statements were developed and translated into criteria that could be used to assess program qualification for LCS. However, the implementation of these policies has not been frequently mandated, and these policies require significant resources. These factors may contribute to LCSPs being of highly variable quality, imaging centers conducting LCS outside of a LCSP, or imaging centers capable of LCS not providing LCS at all.

We aimed to determine the presence of recommended structural and quality elements within established LCSPs and determine institutional barriers to performing LCS within a community-based, multistate healthcare network.

## 2. Methods

We conducted a cross-sectional study using two structured telephonic interviews of LCSP navigators and imaging center administrators within a United States community-based healthcare network between 1 June 2018 and 31 July 2020. During the study period, the healthcare network spanned six states (Alaska, California, Montana, Oregon, Texas, and Washington) with 24 LCSPs located in varied healthcare settings associated with 90 inpatient and outpatient imaging centers with access to a low-dose computed tomography (LDCT) scanner. Twenty two (92%) of the 24 established LCSPs were active at the time of interview and were thus included. Of the 90 imaging centers, 76 (84%) imaging center administrators were successfully contacted and interviewed. The Institutional Review Board determined this study included nonpatient research.

### 2.1. Interviews

**Figure 1:**
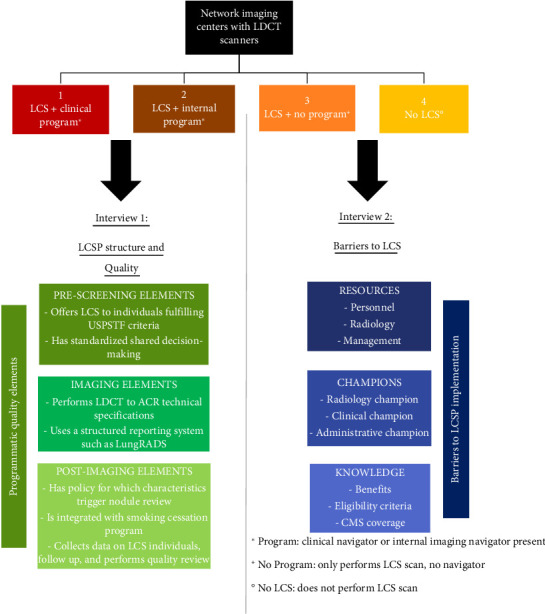
Components of the two interviews addressing lung cancer screening program structure and quality, and barriers to lung cancer screening (ACR, American College of Radiology; LCS, lung cancer screening; LCSP, lung cancer screening program; LDCT, low-dose computed tomography; Lung-RADS, lung imaging reporting and data system; USPSTF, United States Preventive Services Task Force).

Two separate interviews were created (Figure 1). Interview 1 (Appendix 1) was delivered to LCSP navigators and was designed to determine the presence of recommended structural and quality elements within LCSPs. This interview included additional questions pertaining to program resources and time commitments. Interview 2 (Appendix 2) was delivered to imaging center administrators and was designed to determine barriers to LCS implementation. This interview addressed three main domains of LCS: resources (personnel, radiology, and management), champions (clinical and administrative), and knowledge (benefits, guidelines, and coverage).

### 2.2. Definitions

A centralized LCSP was defined by shared decision-making (SDM), evaluation, and management occurring at a single central site. A decentralized LCSP was defined by SDM, evaluation, and management provided by multiple providers across geographically diverse community care settings.

An outpatient imaging center was defined as a free-standing center where only outpatient imaging was provided. An inpatient imaging center was defined as a hospital imaging department which provided both inpatient and outpatient imaging services.

### 2.3. Statistical Analysis

Descriptive data were summarized as counts and percentages.

## 3. Results

### 3.1. LCSP Structure and Quality

#### 3.1.1. Overview

Across the 22 active LCSPs, 5 slightly differing logistical program flows were identified (Appendix 3). The main differences included a centralized versus decentralized program structure, SDM timing, program coordinator versus diagnostic imaging technician scheduler, LungRADS category follow-up, and attrition management. All programs ceased LCS when individuals became ineligible.

#### 3.1.2. Structural Elements

Individuals were identified to be eligible for LCS by their primary care physicians (PCPs) office in the majority (18, 82%) of LCSPs; however, in 3 (14%) programs, this was accomplished by a wellness best practice alert in the electronic health record, and in 1 (4%) program, it was unknown.

A decentralized program structure was reported in 20 (91%) of the LCSPs, where 15 (75%) had referrals purely from PCPs and 5 (25%) had referrals from PCPs as well as pulmonologists and oncologists. Within these decentralized LCSPs, the eligibility for LCS was confirmed by the referring provider in 9 (45%), purely by the order form in 7 (35%), and by a combination of the referring provider, the order form, and/or the program coordinator in 4 (20%).

A centralized program structure was reported in 2 (9%) of the LCSPs, one of the programs had referrals purely from PCPs, and the other had referrals from PCPs as well as pulmonologists and self-referrals. In both centralized programs, the eligibility of the individual was confirmed by the scheduler.

#### 3.1.3. Patient and Provider Education

Navigators of all the programs (22, 100%) understood the aim and importance of SDM and its documentation. In all 20 decentralized programs, SDM occurred at the initial PCP visit. However, only 1 (5%) program utilized standardized SDM tools. In the 2 centralized programs, SDM occurred at the first office visit with the nurse practitioner. In one of the programs, this was the same day as the LDCT scan, and in the other, it was on a separate day. In both centralized programs, standardized SDM tools were utilized.

Fourteen (64%) of the navigators reported facilitated education of participating and referring providers through meetings, electronic, or paper information dissemination and other resources.

#### 3.1.4. Radiology Characteristics

Eighteen (82%) navigators reported utilizing a single radiology group, while 4 (18%) navigators utilized multiple radiology groups. Twenty-one (95%) navigators reported utilizing a specific group of LCS radiologists, while 1 (5%) navigator was unsure. In addition, 20 (90%) navigators reported they coordinated with their radiology group to ensure radiologists were trained on LungRADS and LCS, 1 (5%) navigator did not coordinate with their radiology group, and 1 (5%) navigator was unsure.

#### 3.1.5. Management Algorithms

Thirteen (59%) LCSPs had a multidisciplinary nodule review (including both centralized programs), of which the following specialties were represented: 12 (92%) included radiology, 12 (92%) included medical oncology, 12 (92%) included radiation oncology, 11 (85%) included pulmonary medicine, and 11 (85%) included thoracic surgery. In addition, 9 (69%) of these 13 LCSPs utilized standard criteria for including an individual's case for review: 7 (78%) included only individuals with LungRADS 4 and 2 (22%) included a combination of LungRADS 4, a nodule larger than 8 mm, and/or a nodule with concerning features.

Twenty (91%) of the LCSPs had the ability to characterize concerning nodules through further imaging, nonsurgical, and surgical approaches without the need for referral.

#### 3.1.6. Data Collection and Review

Navigators from 21 (95%) of the 22 LCSPs reported that they collected patient information for external purposes, such as reporting to the ACR registry and/or an oversight body. In 12 (57%) of these 21 LCSPs patient information was collected automatically, while in 9 (43%), it was collected manually. Of these navigators, 19 (90%) had access to the collected patient information.

Navigators from 15 (68%) of the 22 LCSPs reported they collected patient information for internal purposes. Fourteen (93%) navigators manually did this, 9 (64%) using Excel and 5 (36%) using other platforms. Data elements commonly collected included number of individuals, demographics, smoking history, cessation, LungRADS category, downstream testing, downstream results, and attrition. The purpose for internal patient information collection was tracking in 12 (80%) of the LCSPs, business/administrative in 2 (13%), and research in 1 (7%).

Nine (41%) of the 22 LCSPs reviewed their collected patient information of which 7 (78%) had no review schedule, 1 (11%) reviewed weekly, and 1 (11%) reviewed monthly, 4 (18%) had an official internal quality review/monitoring process (all headed by the navigator), and 2 (9%) were integrated with a research committee.

The majority (15/22, 68%) of navigators spent up to 2 h per day on the abovementioned tasks, 4 (18%) spent 2–4 h per day, and 3 (14%) spent more than 4 h per day. For the 17 (77%) navigators whose role also included clinical care, 2 (12%) reported that charting and documenting for their LCSP took up more time than they spent on clinical care, 5 (29%) spent roughly equal time on both, and 10 (59%) reported they spent less time on LCSP administrative duties than clinical care.

#### 3.1.7. Smoking Cessation

Ten (45%) of the LCSPs utilized a standard procedure for smoking cessation and/or counseling: 4 (40%) referred to a quit line, 3 (30%) used face-to-face counseling, and 3 (30%) used other or a combination of methods. Of the 22 LCSPs, 5 (23%) navigators reported their cessation counselors were Certified Tobacco Treatment Specialists (CTTSs) (both centralized programs utilized face-to-face counseling with a CTTS).

#### 3.1.8. Challenges

The biggest challenges reported by navigators in the operation of their LCSP in their final concluding statement included limited LDCT scanner capacity, lack of referring provider education, manual tracking burden/lack of technology infrastructure, and funding.

### 3.2. Barriers to LCS Implementation

#### 3.2.1. Overview

Forty five (59%) of the 76 imaging administrators interviewed had a LCSP associated with their imaging site, while 31 (41%) did not.

Of these 31 imaging sites, 8 (26%) were performing LCS. Fourteen (45%) of the imaging sites were inpatient, none of which performed LCS; while 17 (55%) were outpatient, 8 (47%) of which performed LCS. Twenty (65%) sites were affiliated with a cancer center or institute.

#### 3.2.2. Personnel

Of the 31 sites, 26 (84%) received general imaging orders from both affiliated providers within their institution and outside providers, whereas 5 (16%) received orders from affiliated providers only. Fifteen (48%) sites received LCS orders, of which 8 (53%) fulfilled those orders. Ten (32%) sites received orders for noncontrast LDCT scans not specifying LCS, of which 6 (60%) sites fulfilled those orders.

#### 3.2.3. Radiology

All 31 sites used LDCT scanners, and all except 1 site had scanners accredited by the ACR. Only 2 (25%) of the 8 sites performing LCS scans reported these to the ACR. Thirty (97%) sites fulfilled requirements for LCS to be covered by CMS, including radiologists involved with the supervision/interpretation of at least 300 chest CTs in the past 3 years and being up to date with the ACR continuing medical education requirements.

#### 3.2.4. Management

Five (16%) sites had a dedicated LCS coordinator/lead and the resources to manage follow-up imaging. However, no sites had the resources to manage incidental findings. Nineteen (61%) sites had the resources to fulfill or maintain a dramatic increase in orders for LCS and all 31 (100%) had access to an electronic medical record.

#### 3.2.5. Champions

Nine (29%) sites had a primary individual who could serve as a radiology champion for LCS, 6 (19%) had a potential affiliated clinical champion, and 10 (32%) had a potential administrative champion. Twelve (39%) sites were involved with a specific specialty that could be responsible for leading a LCSP.

#### 3.2.6. Knowledge

Thirty (97%) of the imaging site administrators interviewed were aware of the benefits of LCS, 23 (74%) were aware of the 2014 USPSTF's eligibility criteria, and 26 (84%) were aware of CMS coverage for LCS.

#### 3.2.7. Specific Barriers

**Figure 2:**
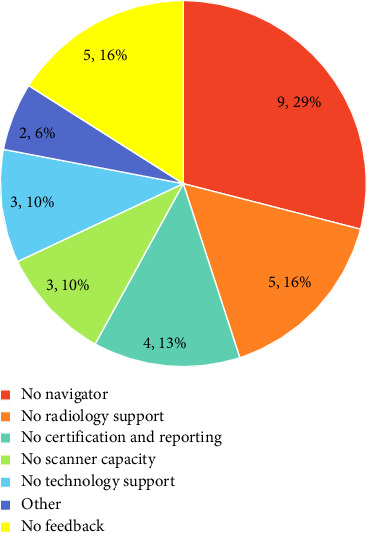
Pie chart stratifying barriers to lung cancer screening program implementation encountered by imaging center administrators.

When asked which requirements would be needed to implement a LCSP at their site, administrators stated predominantly resources (11, 35%), resources and champions (6, 19%), and predominantly champions (2, 6%). Specific barriers are shown in Figure 2.

## 4. Discussion

This is one of the first studies evaluating the implementation of recommendations for components necessary for a high quality LCSP. The study identified a wide variation in the structural elements, quality components, and resource allocation of LCSPs within our network. Notably, few programs incorporated standardized SDM tools, collected comprehensive data on enrolled individuals, performed a quality review, and/or utilized a smoking cessation program. In addition, the majority of LCSP navigators felt the need to collect additional data for tracking purposes. Of the imaging sites within the network, almost half were not associated with a LCSP. A lack of navigators, resources for follow-up, and champions were identified as institutional barriers to LCSP implementation.

Historically, CMS and insurance providers have dictated the logistical flow of LCS. However, many institutions do not audit the quality of established LCSPs, and there is currently no standardized template for planning, implementing, and/or maintaining a LCSP, although several recommendations have been published [13–16]. This leaves each institution to design a program best fitted to their available resources and local environment, resulting in differing program structures and subsequent variation in program quality [17–19]. The predominant logistical difference identified within our network was a centralized versus decentralized program structure. Centralized programs appeared to more commonly use standardized SDM tools, multidisciplinary nodule review boards, follow-up pathways, and dedicated smoking cessation resources; while decentralized programs covered a wider geography. Previous literature supports this, with a qualitative study of clinicians participating in LCS identifying structured centralized LCS to be essential to a LCSP's success, and a randomized controlled trial identifying implementation of a patient navigation program in community health centers to significantly increase LCS [20, 21]. In contrast, strict formalized program structures and intensive resource requirements may prevent sites from initiating LCS.

While most navigators within our network collected data for reporting to the ACR and/or an oversight body, half of them identified their current tools to be insufficient and felt the need to maintain separate databases to aid internal tracking purposes. Lack of informatics support at all steps in the LCS process, from eligibility determination to tracking and communication, has also been reported in multiple previous studies [16, 20]. This highlights the importance of efficient resource development and allocation, not only to increase the quality and streamline current LCS practices but also to allow for an increase in volumes as eligibility evolves and expands [10, 11, 22].

The presence of quality elements has real implications for the experiences and outcomes of LCSP participants. In this study, less than half of the LCSPs utilized a standard procedure for smoking cessation and only five programs utilized a CTTS. Smoking cessation is arguably the most vital component of LCS because of its significant reduction in lung cancer-related mortality. A secondary analysis of the NLST identified a 7-year smoking abstinence to reduce lung cancer-specific mortality at a magnitude comparable with LCS, and an even greater reduction when abstinence was combined with LCS [23]. This emphasizes the need for vigorous smoking cessation efforts to be coupled with LCSPs.

We identified a large opportunity (contingent on additional available resources) to increase LCS within our network as 40% of affiliated imaging sites were not associated with a LCSP, even though they were associated with a cancer center. In addition, a quarter of these sites were performing some LCS scans; however, the management of these scanned individuals was not being regulated. Even with imaging site administrators stating they could maintain a heightened workload, the type of program and staffing play a key role in the available resources required to implement and maintain quality LCS. Provider's lack of time, knowledge, and counseling expertise has been widely identified and documented, particularly for preventative care and in decentralized programs, where a study reported the mean time discussing LCS to be less than a minute [20, 24–27].

Solutions for some of these issues have been proposed and trialed. Since LCS does not solely comprise an imaging study, a multidisciplinary programmatic approach will likely yield the highest quality results. Time constraints of PCPs in decentralized LCSPs may be alleviated by certain clinical tasks (SDM and education) being performed by other trained health care professionals; and since the majority of LCS referrals originate in the PCPs office, tools to assist clinicians in identifying eligible individuals (best practice alerts) and to streamline ordering and tracking would not only increase LCS uptake but could also advance decentralized LCSPs [28]. Efforts to do so by some have shown success. A 1-year pilot LCS implementation project in a large academic primary care clinic where systematic processes to identify eligible individuals, offer LCS, conduct SDM, and order scans was developed demonstrated a substantial increase in smoking history collection and documentation of SDM [18]. The same group also operationalized a system of standardized reporting and follow-up pathways, with > 90% fidelity across subspecialities and PCPs [19].

While this study was designed to be descriptive, there are limitations in the generalizability of the findings as the interviews were conducted within a single healthcare system in the United State, and other systems and settings may differ.

## 5. Conclusions

In conclusion, we identified a wide variation in the structure, quality, and resource allocation within our network of LCSPs. Program type and staffing may play a role in the resources available for implementation. Further research identifying the implications this variation has on outcomes, operational cost, and experience may shed light on whether stringent program quality control is needed and which quality elements are essential.

## Data Availability

The data that support the findings of this study are available from the corresponding author upon reasonable request.

## Supporting Information

Appendix 1. Interview 1 provided to lung cancer screening program navigators: Lung cancer screening program structure and quality.

Appendix 2. Interview 2 provided to imaging center administrators: Barriers to lung cancer screening.

Appendix 3. Lung cancer screening program logistical flow pathways (Lung-RADS, lung imaging reporting, and data system).
