# Supplementary material for: Community-Based Lung Cancer Screening Program Structure, Quality, and Barriers: The Struggle for Implementation
Source: Can Respir J. 2025 Mar 21;2025:9683951. doi: 10.1155/carj/9683951 (PMC11952916; doi:10.1155/carj/9683951)
Supplement: Supporting Information — Additional supporting information can be found online in the Supporting Information section. [file 9683951.f1.docx]

**APENDICES**

**Appendix 1.** Interview 1: Lung cancer screening program structure and quality

- Overview:
  1. Please describe the flow of your program from referral up to the first CT scan.
  2. Please describe what would happen for two different possible results from the first scan for each of these patients (from the POV of LCSP, not clinical care)
     1. LungRADS 1&2
     2. LungRADS 3&4
     3. Describe follow-up procedure if patient didn’t show for appointment.
     4. Are attempts to follow-up for LungRADS 1/2 is the same for 3/4?

Same / Different

- Data collection/analysis/manipulation: Now we’re going to dive into specifics concerning data collection and quality review, to better understand your data infrastructure.

1. Do you collect data from your LCSP for *external* purposes, for example reporting to ACR registry and/or an outside oversight body?

Yes collect/report to ACR

Yes collect/report to other

No

- 1. How/where is it collected? Manual/Automatic
  2. Do you have access to collected data? Yes/No

1. Do you use multiple radiology groups? Yes/No
   1. If so, is this true of all groups? Same/Different
2. Do you collect data for *internal* purposes only? (Distinguish clinical from research – do you collect data other than the ACR data for your own purposes?)

Yes/No

- 1. How/where is it collected? Manual/Automatic

Where?

- 1. Do you have access to collected data? Yes/No
  2. What data?
  3. What purpose? Why these data elements specifically?

1. Does your program review collected data? Schedule?

Yes / No schedule / No review

1. Who accomplishes the above tasks for your program?
2. How much time is spent on those tasks per day?
3. Does charting and documenting for LCS take up more, less than, or equal to the amount of time spent on actual clinical care? More / Less / Equal

- Quality initiatives, LCSP policies for reviewing data

1. Does your program have an internal quality review or monitoring process, and what does it entail? *Quality initiatives could be set by either the LCSP or its supervising body. Topics to review could include: attrition rates, number and characteristics of diagnosed lung cancers, number of biopsies or surgeries as a result of screening, number of complications as a result of diagnostic tests or procedures, enrolment of minorities in LCS, participation in smoking cessation programs, etc.*

Yes / No

Robust / Middle / Weak

1. Who accomplishes the above tasks for your program?
2. Does your program have or work with a research committee? Yes / No

- Structural and ‘flow’ elements: Now, these are some questions concerning specific elements in your LCSP

1. Does your program participate in the ACR Registry (for CMS reimbursement)?

Yes / No

- 1. Find online: designated lung cancer screening center (from the ACR)?

Yes / No

1. Describe your program structure in terms of centralized / hybrid / decentralized

1. If centralized, who refers patients to the facility, and how/is their eligibility ascertained before being seen?

Who can refer?

Determination of eligibility

1. If decentralized, who refers patients to the facility, and how/is their eligibility ascertained before being seen?

Who can refer?

Determination of eligibility

- *Patient/Provider* *Education*

1. Does your LCSP coordinate with the radiology group to ensure radiologists are trained on LungRADS and LCS? Yes/No
2. Do you have a specific group of radiologists who will do LCS? Yes/No
3. Does your LCSP facilitate education of participating/referring providers, i.e. through grand round meetings, electronic or paper information dissemination, etc.?

Yes/No

Description

1. How does your program identify patients eligible for LCS?

EHR / Best practice advisory bubble (BPA) / hard stop eligibility to place order / primary care offices

1. Your program has _____ conduct shared decision making. At what point of the process is shared decision making done?

With Primary care / first visit to office (same at CT) / first visit (different from CT)

1. Do you use standardized shared decision-making tools?

Yes, in clinic / Yes, send home with instructions / No, conversation only

1. For your program, what is the aim of shared decision making?

- *Lung Nodule Management Algorithms*

1. You reported you __ do or don’t __ have a multidisciplinary nodule review, correct?

Yes/No

- - 1. Are the following represented
       1. Radiology Yes/No
       2. Pulmonary medicine Yes/No
       3. Thoracic surgery Yes/No
       4. medical oncology Yes/No
       5. Radiation oncology Yes/No

1. Do you have a standard to decide whether to bring patients to board review? What is it based on? Yes/No

Size of nodule / LungRADS / other

1. Do you “Have ability to characterize concerning nodules through PET imaging, nonsurgical, surgical approaches?” Or would you need to refer

Yes/No

1. Do you collect data concerning the course of care of patients, such as number of scans, biopsies, procedures, and complications?

Yes/No

1. *Who takes ownership of abnormal findings on the first LCS exam, including referral to a specialist? On subsequent exams?
2. How do you deal with Incidental Findings?

- *Smoking Cessation*

1. What is your standard procedure, if any, regarding smoking cessation and/or counseling in your LCSP?

Face to face / prescribing / refer for cessation / refer to quit line

1. Are the people who do counseling certified tobacco treatment specialists (CTTS)? Yes/No
2. Do you collect data on smoking cessation and interventions by your program?

Yes/No

- *How often/Long to Screen*

1. Please describe how or if your LCSP structure ensures follow-up
2. Do you ever receive feedback that patients feel harassed by the follow-up mechanism?

Yes/ No feedback of harassment

1. What is your policy for how often/ how long to screen (insurance may mandate keeping with USPSTF guidelines)
   - 1. in case of LungRADS 1,2
     2. in case of LungRADS 3,4
2. What is your biggest challenge in LCS, what would you want to communicate to those who make guidelines, as someone who is actually “on the ground”?
3. Who is radiology contact?

**Appendix 2.** Interview 2: Barriers to lung cancer screening

1. Describe your location’s radiology/imaging center.

(outpatient vs. inpatient, location etc.)

1. Does your site conduct lung cancer screening?

☐YES ☐NO

1. Is your site affiliated with a lung cancer screening program?

☐YES ☐NO

Coordinator for program:

If no program, internal LCS imaging coordinator:

# *Resources*

## Personnel

1. Is your site affiliated with any specific specialties or independent specialty groups?

☐YES ☐NO

*If yes, please list.*

1. Is your site affiliated with a Cancer Center or Institute?

☐YES ☐NO

*If yes, please list.*

1. Does your site receive orders only from affiliated providers within the institution or do you receive outside orders from private clinicians/groups?

☐YES (both) ☐NO (only affiliated)

1. Does your site receive lung cancer screening orders? (marked LCS or non-contrast LDCT?)

☐YES ☐NO

1. Do you turn away these LCS/LDCT orders or carry them out?

☐YES (turn away) ☐NO (carry them out)

## Radiology

1. Does your site’s radiology department use low-dose CT?

☐YES ☐NO

1. Does your site’s radiology department report to the American College of Radiology?

☐YES ☐NO

1. If yes to 10, what specific modalities such as MRI, CT or other types of screening such as breast imaging do you report?
2. Are your CT scanners accredited by the ACR? (this is the first step in becoming an ACR Lung Cancer Screening Center)

☐YES ☐NO

1. For LDCT screening to be covered by CMS, the interpreting radiology physician must have been involved with the supervision and interpretation of at least 300 chest CTs in the past three years and be up-to-date with the ACR continuing medical education (CME) requirements. Do you have personnel at your site that meet both of these requirements?

☐YES ☐NO

Management

1. Do you have an individual dedicated to managing LCS patients?

☐YES ☐NO

1. Does your site have the resources to manage follow-up imaging with patients?

☐YES ☐NO

1. Does your site have the resources to manage incidental findings?

☐YES ☐NO

1. If there was a dramatic increase in orders for LCS, would your site have the resources to fulfill/maintain this heightened workload?

☐YES ☐NO

1. Does your site have access to an electronic medical record?

☐YES ☐NO

# *Champions*

1. Is there a primary person who could serve as a radiology champion for **LCS?**

☐YES ☐NO

Name:

Contact info:

1. Is there a primary person who could serve as an affiliated clinical champion for **LCS?**

☐YES ☐NO ☐Same as above

Name:

Contact info:

1. Is there a primary person who could serve as the administrative champion for **LCS?**

☐YES ☐NO ☐Same as above

Name:

Contact info:

1. Is there a specific specialty involved with your site that would most likely be responsible for leading a **LCSP?**

☐YES ☐NO

Med Onc: ___

Pulmonology: ___

Thoracic surgery: ___

Radiology: ___

PCPs: ___

# *Knowledge*

## Learned knowledge

1. Are you aware of the benefits of lung cancer screening?

(NLST 2011, screening for individuals at high risk reduces lung cancer mortality by 20%)

☐YES ☐NO

1. Are you aware of the U.S. Preventive Services Task Force (USPSTF) Grade B eligibility criteria for lung cancer screening enacted in 2013?

(55-80 y.o., 30 pack year smoking history, currently smoke or quit in last 15 years)

☐YES ☐NO

1. Are you aware that the Centers for Medicare and Medicaid Services covers lung cancer screening for patients who meet the USPSTF guidelines?

☐YES ☐NO

1. What do you think would be required to start a lung cancer screening program at your site?
2. What are the anticipated barriers to starting a lung cancer screening program at your site?
3. What is your biggest need or barrier to more efficiently conducting LCS at your site?

**Appendix 3.** Lung cancer screening program logistical flow pathways (Lung-RADS, lung imaging reporting and data system).

**Program Flow 1 – Centralized Program (n=1)**

**FOLLOW-UP PROCESS**

**Program Coordinator tracks individual to ensure follow-up and treatment**

- Electronic medical record recall configured for appropriate timeline

- 1 month before appointment, up to 2 phone calls to schedule

- If no contact, electronic letter

- If no contact, paper letter and close out of program

**Program Flow 2 – Centralized Program (n=1)**

**FOLLOW-UP PROCESS**

**Program Coordinator tracks individual to ensure follow-up and treatment**

- Electronic medical record recall configured for appropriate timeline

- 1 month before appointment, up to 2-3 phone calls to schedule

- If no contact, electronic letter

- If no contact, paper letter and close out from program

**FOLLOW-UP PROCESS**

**Program Coordinator tracks individual to ensure follow-up and treatment**

- Electronic medical record (n=6) and diagnostic imaging (n=1) recall configured for appropriate timeline

- If no contact (3 calls/letter), calls provider (n=5)

- If still no contact, closes out after 1 year (n=1)

**Program Flow 3 – Decentralized Programs (n=6)**

**FOLLOW-UP PROCESS**

**Program Coordinator tracks individual to ensure follow-up and treatment**

- Tracking system recall configured for appropriate timeline (n=1), manual by Program Coordinator (n=2)

- If no contact 1 call and/or 1 letter, if no response closed out (n=2), except LungRADS 4 where another call occurs (n=1)

- No procedure for no contact, if LungRADS 1-2 then closed out (n=1)

**Program Flow 4 – Decentralized Programs (n=3)**

**FOLLOW-UP PROCESS**

**Program Coordinator tracks individual to ensure follow-up and treatment**

- Electronic medical record/tracking system and diagnostic imaging recall configured for appropriate timeline

- If no contact 3 letters sent, then closed out (n=2), except LungRADS 3-4 where only closed out after another call (n=1)

- If no contact, referring provider contacted once (n=1)

- No standard procedure (n=1)

**Program Flow 5 – Decentralized Programs (n=4)**
